# Determinants within the *C*-Terminal Domain of *Streptomyces lividans* Acetyl-CoA Synthetase that Block Acetylation of Its Active Site Lysine *In Vitro* by the Protein Acetyltransferase (Pat) Enzyme

**DOI:** 10.1371/journal.pone.0099817

**Published:** 2014-06-11

**Authors:** Alex C. Tucker, Jorge C. Escalante-Semerena

**Affiliations:** Department of Microbiology, University of Georgia, Athens, Georgia, United States of America; University Paris Diderot-Paris 7, France

## Abstract

Reversible lysine acetylation (RLA) is a widespread regulatory mechanism that modulates the function of proteins involved in diverse cellular processes. A strong case has been made for RLA control exerted by homologues of the *Salmonella enterica* protein acetyltransferase (*Se*Pat) enzyme on the broadly distributed AMP-forming CoA ligase (a.k.a. acyl-CoA synthetases) family of metabolic enzymes, with acetyl-CoA synthetase (Acs) being the paradigm in the field. Here we investigate why the Acs homologue in *Streptomyces lividans* (*Sl*Acs) is poorly acetylated *in vitro* by the *S. lividans* protein acetyltransferase (*Sl*Pat) enzyme. Chimeras of *S. enterica* Acs (*Se*Acs) and *S. lividans* Acs (*Sl*Acs) constructed during the course of this work were acetylated by *Sl*PatA *in vitro*, retained most of their activity, and were under RLA control in a heterologous host. We identified *Se*Acs residues *N*- and *C*-terminal to the target lysine that when introduced into *Sl*Acs, rendered the latter under RLA control. These results lend further support to the idea that Pat enzymes interact with extensive surfaces of their substrates. Finally, we suggest that acetylation of *Sl*Acs depends on factors or conditions other than those present in our *in vitro* system. We also discuss possible explanations why *Sl*Acs is not controlled by RLA as defined in other bacterial species.

## Introduction

Reversible lysine acetylation (RLA) is a post-translational modification that occurs in all domains of life [1] and affects diverse cellular processes and functions. Acetyltransferases transfer the acetyl moiety from acetyl-CoA to the ε-amino group of the target lysine. Lysine acetylation can affect enzyme activity [2], protein stability [3], protein-protein interactions, or DNA binding [4]. Yeast Gcn5 protein (yGcn5p)-related *N*-acetyltransferases (a.k.a., GNATs), classified by amino acid sequence and structure [5], are the only class of acetyltransferases found in all domains of life [6]. GNATs were first identified for their role in modification of histones [7]. Crystal structures and biochemical analyses of the yGcn5p, the founding member of the GNAT family, with representative peptides from histones has provided valuable information about the substrate specificity and substrate recognition by GNATs [8], [9].

Members of the GNAT family also acetylate metabolic enzymes. For example, in *Salmonella enterica*, the enzyme acetyl-CoA synthetase (*Se*Acs) is acetylated by the protein acetyltransferase (*Se*Pat), a two-domain acetyltransferase that contains a large domain of unknown function and a *C*-terminal GNAT domain [10]. *Se*Acs is a member of the AMP-forming CoA ligase family of enzymes that converts carboxylic acids to their CoA thioesters via an acyl-AMP intermediate [11]. Acetylation of the active site lysine of AMP-forming CoA ligases prevents the adenylylation of the carboxylic acid. In addition to Pat from *S. enterica*, GNATs are known to acetylate members of the of AMP-forming CoA ligase family (including Acs) in *Rhodopseudomonas palustris* [12], [13], *Bacillus subtilis* [14], and *Mycobacterium smegmatis* [15]. The Acs homologue from *Streptomyces coelicolor* is acetylated *in vivo* [16], but the GNAT responsible for acetylation of *S. coelicolor* Acs is unknown.

Knowledge of the interactions of GNAT with their proteins substrates is limited. *R. palustris* encodes a single-domain GNAT (*Rp*KatA) and a homologue of the *Se*Pat GNAT (*Rp*Pat). *Rp*KatA and *Rp*Pat discriminate among members of the AMP-forming CoA ligase family produced by *R. palustris* [13]. In addition to the target lysine, *Rp*Pat recognizes a loop greater than 20 Å from the target lysine, suggesting that Pat enzymes interact with a large surface of the acceptor substrate [17]. As a proof of principle, the introduction of this recognition loop into *R. palustris* methylmalonyl-CoA mutase (*Rp*MatB), an AMP-forming CoA ligase that is not a substrate of *Rp*Pat, rendered *Rp*MatB a target of acetylation by *Rp*Pat. Thus, synthetic chimeras of AMP-forming CoA ligases have yielded valuable information about how GNATs recognize protein substrates and have produced AMP-forming CoA ligases that are placed under the regulation of lysine acetylation.

*Rp*Pat and *Se*Pat enzymes acetylate their cognate Acs proteins. Although the GNAT responsible for the acetylation of Acs in *S. coeolicolor* is unknown, the closely related actinomycete *Streptomyces lividans* encodes *Sl*PatA, a two-domain homologue of *Se*Pat and *Rp*Pat enzymes. Significantly, *Sl*PatA does not efficiently acetylate the *S. lividans* Acs (*Sl*Acs) *in vitro* [18], making this the first Acs enzyme that is not efficiently acetylated by a Pat acetyltransferase. In contrast, *Sl*PatA efficiently acetylates *Se*Acs. Here we probe the amino acid sequences in *Se*Acs that rendered it a better substrate for *Sl*PatA than *Sl*Acs is. By replacing amino acids from *Se*Acs into the *C*-terminus of *Sl*Acs, we constructed *Sl*Acs-*Se*Acs chimeras that were efficiently acetylated by *Sl*PatA. One *Sl*Acs-*Se*Acs chimera contained 41 amino acid differences from *Sl*Acs. As a result of these changes, the *Sl*Acs-*Se*Acs chimera was subject to regulation by *Sl*PatA. We used a heterologous model system to demonstrate that the *Sl*Acs-*Se*Acs chimera was subject to RLA regulation *in vivo* by *Sl*PatA. In sum, we identified regions in *Se*Acs that were critical for recognition by *Sl*PatA, and transferring of these residues into the poor substrate *Sl*Acs resulted in a *Sl*Acs variant that was efficiently regulated by *Sl*PatA.

## Materials and Methods

### Bacterial Strains and Growth Conditions

All strains and plasmids used in this study are listed in Tables 1 and 2, respectively. *Escherichia coli* and *Salmonella enterica* strains were grown at 37°C in lysogeny broth (LB, Difco) [19] or no-carbon essential (NCE) minimal medium [20] supplemented with sodium acetate (10 mM), MgSO_4_ (1 mM), and ampicillin (100 µg ml^−1^). When necessary, antibiotics were used at the following concentrations: ampicillin, 100 µg ml^−1^; tetracycline, 10 µg ml^−1^; chloramphenicol, 12.5 µg ml^−1^, kanamycin, 50 µg ml^−1^. L-(+)-arabinose was added at varying concentrations (5 or 200 µM) to induce the expression of *S. enterica acs*, *S. lividans acs*, and *acs* chimeras cloned into the expression vector pBAD30 [21]. Isopropyl β-D-1-thiogalactopyranoside (IPTG) was added to a final concentration of IPTG (0–500 µM) to induce expression of *S. lividans patA* (EFD66247) clones into the expression vector pSRK-Km [22]. Growth experiments were performed at 37°C using a microtiter plate and a microtiter plate reader (Bio-Tek Instruments). All growth data are plotted as the mean of three data points.

**Table 1:** Strains used in this study.

Strain	Relevant Genotype and description	Source
***S. enterica strains***		
TR6583	*metE205 ara-9*	K. Sanderson via J. Roth
**Derivatives of TR6583**		
JE9152	*metE205 ara-9* Δ*acs2* Δ*cobB1330 pat1*:: Tn*10*d(*tet^+^*)	Laboratory Collection
JE9894	*metE205 ara-9* Δ*acs2 pat1*:: Tn*10*d(*tet^+^*)	Laboratory Collection
JE13238	*metE205 ara-9* Δ*acs2* Δ*pta127*	Laboratory Collection
**Derivatives of JE9152**		
JE18793	*metE205 ara-9* Δ*acs2* Δ*cobB1330 pat1*:: Tn*10*d(*tet^+^*)/pBAD30 pSRK-Km	This work
JE18794	*metE205 ara-9* Δ*acs2* Δ*cobB1330 pat1*:: Tn*10*d(*tet^+^*)/pBAD30 p*Sl*PatA9	This work
JE18795	*metE205 ara-9* Δ*acs2* Δ*cobB1330 pat1*:: Tn*10*d(*tet^+^*)/p*Sl*Acs47 pSRK-Km	This work
JE18796	*metE205 ara-9* Δ*acs2* Δ*cobB1330 pat1*:: Tn*10*d(*tet^+^*)/p*Sl*Acs47 p*Sl*PatA9	This work
JE18797	*metE205 ara-9* Δ*acs2* Δ*cobB1330 pat1*:: Tn*10*d(*tet^+^*)/p*Sl*Acs48 pSRK-Km	This work
JE18798	*metE205 ara-9* Δ*acs2* Δ*cobB1330 pat1*:: Tn*10*d(*tet^+^*)/p*Sl*Acs48 p*Sl*PatA9	This work
JE18799	*metE205 ara-9* Δ*acs2* Δ*cobB1330 pat1*:: Tn*10*d(*tet^+^*)/pACS59 pSRK-Km	This work
JE18800	*metE205 ara-9* Δ*acs2* Δ*cobB1330 pat1*:: Tn*10*d(*tet^+^*)/pACS59 p*Sl*PatA9	This work
**Derivatives of JE9894**		
JE18801	*metE205 ara-9* Δ*acs2 pat1*:: Tn*10*d(*tet^+^*)/pBAD30 pSRK-Km	This work
JE18802	*metE205 ara-9* Δ*acs2 pat1*:: Tn*10*d(*tet^+^*)/pBAD30 p*Sl*PatA9	This work
JE18803	*metE205 ara-9* Δ*acs2 pat1*:: Tn*10*d(*tet^+^*)/p*Sl*Acs47 pSRK-Km	This work
JE18804	*metE205 ara-9* Δ*acs2 pat1*:: Tn*10*d(*tet^+^*)/p*Sl*Acs47 p*Sl*PatA9	This work
JE18805	*metE205 ara-9* Δ*acs2 pat1*:: Tn*10*d(*tet^+^*)/p*Sl*Acs48 pSRK-Km	This work
JE18806	*metE205 ara-9* Δ*acs2 pat1*:: Tn*10*d(*tet^+^*)/p*Sl*Acs48 p*Sl*PatA9	This work
JE18807	*metE205 ara-9* Δ*acs2 pat1*:: Tn*10*d(*tet^+^*)/pACS59 pSRK-Km	This work
JE18808	*metE205 ara-9* Δ*acs2 pat1*:: Tn*10*d(*tet^+^*)/pACS59 p*Sl*PatA9	This work
**Derivatives of JE13238**		
JE13787	*metE205 ara-9* Δ*acs2* Δ*pta127*/pBAD30 bla^+^	This work
JE14947	*metE205 ara-9 Δacs2* Δ*pta127*/p*Sl*Acs6 bla^+^	This work
***E. coli strains***		
JE9314	C41(λDE3) *pka12*:: *kan^+^*	Laboratory Collection

**Table 2:** Plasmids used in this study.

Plasmid	Genotype	Source or method
pBAD30	P*_araBAD_* expression vector, *bla^+^*	[21]
p*Sl*Acs6	*S. lividans acs^+^* allele (EFD66247) in pBAD30, *bla* ^+^	Standard cloning
p*Sl*Acs47	*S. lividans acs^+^* allele (EFD66247) with *N*-terminal H_6_ tag in pBAD30, *bla* ^+^	Standard cloning
p*Sl*Acs48	*S. lividans acs* –*S. enterica acs* chimera allele with *N*-terminal H_6_ tag in pBAD30, *bla* ^+^	Standard cloning
pACS59	*S. enterica acs^+^* allele with *N*-terminal H_6_ tag in pBAD30, *bla* ^+^	Standard cloning
pSRK-Km	*lacI^q^-lac* promoter-operator expression vector, *kan^+^*	[22]
p*Sl*PatA9	*S. lividans patA* ^+^ allele (EFD66247) in pSRK-Km, *bla^+^*	[18]
pKLD66	*N*-terminal, rTEV-cleavable MBP-His_6_-tag overexpression vector, *bla* ^+^	[25]
pSlAcs7	*S. lividans acs* ^+^ (EFD66247) C-terminal domain (D519-D649) in pKLD66, *bla* ^+^	Standard cloning
pTEV5	*N*-terminal, rTEV-cleavable His_6_-tag overexpression vector, *bla^+^*	[25]
p*Sl*Acs1	*S. lividans acs^+^* allele (EFD68454) in pTEV5, *bla^+^*	[18]
p*Sl*PatA1	*S. lividans patA* ^+^ allele (EFD66247) in pTEV5, *bla^+^*	[18]
pACS38	*S. enterica acs^+^* C-terminal domain (D518-S652) in pTEV5, *bla^+^*	Standard cloning
p*Sl*Acs8	A1 chimera: *Sl*Acs 520 *Se*Acs in pTEV5, *bla* ^+^	Overlap-extension PCR
p*Sl*Acs9	A2 chimera: *Sl*Acs 550 *Se*Acs in pTEV5, *bla* ^+^	Overlap-extension PCR
p*Sl*Acs12	A3 chimera: *Sl*Acs 560 *Se*Acs in pTEV5, *bla* ^+^	Overlap-extension PCR
p*Sl*Acs22	A4 chimera: *Sl*Acs 566 *Se*Acs in pTEV5, *bla* ^+^	Overlap-extension PCR
p*Sl*Acs10	A5 chimera: *Sl*Acs 582 *Se*Acs in pTEV5, *bla* ^+^	Overlap-extension PCR
p*Sl*Acs11	A6 chimera: *Sl*Acs 617 *Se*Acs in pTEV5, *bla* ^+^	Overlap-extension PCR
p*Sl*Acs14	B1 chimera: *Sl*Acs 550–582 *Se*Acs in pTEV5, *bla* ^+^ in pTEV5, *bla* ^+^	Overlap-extension PCR
p*Sl*Acs15	B2 chimera: *Sl*Acs 550–603 *Se*Acs in pTEV5, *bla* ^+^ in pTEV5, *bla* ^+^	Overlap-extension PCR
p*Sl*Acs23	B3 chimera: *Sl*Acs 550–618 *Se*Acs in pTEV5, *bla* ^+^ in pTEV5, *bla* ^+^	Overlap-extension PCR
p*Sl*Acs17	B4 chimera: *Sl*Acs 550–627 *Se*Acs in pTEV5, *bla* ^+^ in pTEV5, *bla* ^+^	Overlap-extension PCR
p*Sl*Acs18	B5 chimera: *Sl*Acs 550–638 *Se*Acs in pTEV5, *bla* ^+^ in pTEV5, *bla* ^+^	Standard cloning
p*Sl*Acs19	B6 chimera: *Sl*Acs 550–643 *Se*Acs in pTEV5, *bla* ^+^ in pTEV5, *bla* ^+^	Standard cloning
p*Sl*Acs26	C1 chimera: *Sl*Acs 550–581 *Se*Acs, 591–627 *Se*Acs in pTEV5, *bla* ^+^	Overlap-extension PCR
p*Sl*Acs27	C2 chimera: *Sl*Acs 550–590 *Se*Acs, 598–627 *Se*Acs in pTEV5, *bla* ^+^	Overlap-extension PCR
p*Sl*Acs28	C3 chimera: *Sl*Acs 550–597 *Se*Acs, 603–627 *Se*Acs in pTEV5, *bla* ^+^	Overlap-extension PCR
p*Sl*Acs29	C4 chimera: *Sl*Acs 550–581 *Se*Acs, 603–627 *Se*Acs in pTEV5, *bla* ^+^	Overlap-extension PCR
p*Sl*Acs44	C5 chimera: *Sl*Acs 615–626 *Se*Acs in pTEV5, *bla* ^+^	Overlap-extension PCR
p*Sl*Acs49	K610A variant of C3 chimera in pTEV5, *bla* ^+^	Site-directed mutagenesis

### Molecular Techniques

DNA manipulations were performed using standard techniques [23]. Restriction endonucleases were purchased from Fermentas. DNA was amplified using Pfu Ultra II Fusion DNA polymerase (Agilent) or Herculase II Fusion DNA polymerase (Agilent). Site-directed mutagenesis was performed using the Quikchange™ Site Directed Mutagenesis kit (Agilent). Plasmids were isolated using the Wizard Plus SV Miniprep kit (Promega) and PCR products were purified using the Wizard SV Gel and PCR Clean-Up System (Promega). DNA sequencing was performed using BigDye® (ABI PRISM) protocols, and sequencing reactions were resolved at the University of Georgia Genomics Facility.

### Plasmids Used for Protein Overproduction

Chimeric proteins encoded by fusing different regions of *S. lividans acs* (EFD68454) and *S. enterica acs* genes were generated by amplifying genomic DNA from *S. lividans* TK24 genomic DNA from *S. enterica* strain TR6583, respectively. Fusion plasmids encoding proteins in which the *N*-terminal domain of *Sl*Acs was fused to the *C*-terminal domain of *Se*Acs at residues 520, 550, 560, 566, 582, 617 were generated by overlap-extension PCR [24], followed by standard cloning into plasmid pTEV5 [25]. Fusion plasmids encoding a protein in which an internal sequence of *Sl*Acs was replaced by the corresponding sequence *Se*Acs were constructed as described below and in Table 2.

Plasmid p*Sl*Acs14 (*Sl*Acs 550–582 *Se*Acs) – the nucleotides encoding the first 582 residues of *Sl*Acs fused to *Se*Acs were amplified from p*Sl*Acs9, fused to the *C*-terminus of *Sl*Acs, and cloned into pTEV5.

Plasmid p*Sl*Acs15 (*Sl*Acs 550–603 *Se*Acs) – the nucleotides encoding the first 603 residues of *Sl*Acs fused to *Se*Acs were amplified from p*Sl*Acs9, fused to the *C*-terminus of *Sl*Acs, and cloned into pTEV5.

Plasmid p*Sl*Acs23 (*Sl*Acs 550–618 *Se*Acs) – the nucleotides encoding the first 618 residues of *Sl*Acs fused to *Se*Acs were amplified from p*Sl*Acs9, fused to the *C*-terminus of *Sl*Acs, and cloned into pTEV5.

Plasmid p*Sl*Acs17 (*Sl*Acs 550–627 *Se*Acs) – the nucleotides encoding the first 627 residues of *Sl*Acs fused to *Se*Acs were amplified from p*Sl*Acs9, fused to the *C*-terminus of *Sl*Acs, and cloned into pTEV5.

Plasmid p*Sl*Acs18 (*Sl*Acs 550–638 *Se*Acs) – the nucleotides encoding the first 638 residues of *Sl*Acs fused to *Se*Acs were amplified from p*Sl*Acs9 and cloned into pTEV5.

Plasmid p*Sl*Acs19 (*Sl*Acs 550–643 *Se*Acs) – the nucleotides encoding the first 643 residues of *Sl*Acs fused to *Se*Acs were amplified from p*Sl*Acs9 and cloned into pTEV5.

Plasmid p*Sl*Acs26 (*Sl*Acs 550–581 *Se*Acs, 591–627 *Se*Acs) – the nucleotides encoding the first 581 residues of *Sl*Acs fused to *Se*Acs were amplified from p*Sl*Acs9 with primers incorporating residues 582–590 from *Sl*Acs, fused to the nucleotides encoding the 64 residues of *Se*Acs fused to *Sl*Acs amplified from p*Sl*Acs17, and cloned into pTEV5.

Plasmid p*Sl*Acs27 (*Sl*Acs 550–590 *Se*Acs, 598–627 *Se*Acs) – the nucleotides encoding the first 590 residues of *Sl*Acs fused to *Se*Acs were amplified from p*Sl*Acs9 with primers incorporating residues 591–597 from *Sl*Acs, fused to the nucleotides encoding the 57 residues of *Se*Acs fused to *Sl*Acs amplified from p*Sl*Acs17, and cloned into pTEV5.

Plasmid p*Sl*Acs28 (*Sl*Acs 550–597 *Se*Acs, 603–627 *Se*Acs) – the nucleotides encoding the first 597 residues of *Sl*Acs fused to *Se*Acs were amplified from p*Sl*Acs9 with primers incorporating residues 598–602 from *Sl*Acs, fused to the nucleotides encoding the 52 residues of *Se*Acs fused to *Sl*Acs amplified from p*Sl*Acs17, and cloned into pTEV5.

Plasmid p*Sl*Acs29 (*Sl*Acs 550–581 *Se*Acs, 603–627 *Se*Acs) – the nucleotides encoding the first 581 residues of *Sl*Acs fused to *Se*Acs were amplified from p*Sl*Acs9 with primers incorporating residues 582–602 from *Sl*Acs, fused to the nucleotides encoding the 52 residues of *Se*Acs fused to *Sl*Acs amplified from p*Sl*Acs17, and cloned into pTEV5.

Plasmid p*Sl*Acs44 (*Sl*Acs 615–626 *Se*Acs) – the nucleotides encoding the first 614 residues of *Sl*Acs were amplified from p*Sl*Acs1, the nucleotides encoding the final 40 residues of *Se*Acs fused to *Sl*Acs amplified from p*Sl*Acs28, and cloned into pTEV5.

The *C*-terminal domain of *Se*Acs was amplified from strain TR6583. DNA fragments were cut with NheI and EcoRI and ligated into pTEV5 [25] cut with the same enzymes. The resulting plasmids directed the synthesis of *Sl*Acs-*Se*Acs chimeras or *Se*Acs *C*-terminal domain (pACS38) each with an *N*-terminal H_6_ tag cleavable by recombinant tobacco etch virus (rTEV) protease prepared as described [26].

The *C*-terminal domain of *Sl*Acs was amplified from *S. lividans* TK24 genomic DNA. The DNA fragments were cut with KpnI and HinDIII and ligated into pKLD66 [25] cut with the same enzymes. The resulting plasmid p*Sl*Acs7 directed synthesis of the *Sl*Acs *C*-terminal domain with an *N*-terminal maltose-binding protein-His_6_ tag cleavable by rTEV protease as described above.

### Construction of Untagged SlAcs Complementation Plasmid

The *S. lividans acs* was amplified from p*Sl*Acs1 with the primers that included an optimized ribosome-binding site. The DNA fragment was cut with EcoRI and HindIII and ligated into pBAD30 [21], cut with the same enzymes. The resulting plasmid p*Sl*Acs6 expresses *Sl*Acs under the control of the P*_araBAD_* promoter.

### Construction of *Se*Acs, *Sl*Acs, and *Sl*Acs

#### Complementation vectors encoding H_6_-tagged *Se*Acs chimera C3

Genes encoding *S. lividans* Acs and the *S. lividans/S. enterica* Acs chimeras were amplified from p*Sl*Acs1 and p*Sl*Acs28, respectively, using primers that included an optimized ribosome-binding site and an *N*-terminal His_6_-tag. *S. enterica acs* was amplified from genomic DNA isolated from JE6583 using primers that included an optimized ribosome-binding site and an *N*-terminal His_6_-tag. The DNA fragments were cut with EcoRI and HindIII and ligated into pBAD30, cut with the same enzymes. The resulting plasmids p*Sl*Acs47, p*Sl*Acs48, and pACS59 produce *Sl*Acs, *S. lividans/S. enterica* Acs chimera C3, and *Se*Acs^WT^, respectively, with His_6_-tags fused *N-*terminal with a Gly-Ser-Gly linker under the control of at the P*_araBAD_* promoter.

#### Purification of *Sl*Acs-*Se*Acs chimeras, *Sl*Acs C-terminal domain, and *Se*Acs C-terminal domain

Plasmids encoding tagged proteins were transformed with pRARE2 (EMD Millipore) into a Δ*pka* derivative of *E. coli* strain C41λ(DE3) [27] (JE9314) to prevent acetylation prior to overproduction. The resulting strains were grown overnight and sub-cultured 1∶100 (v/v) into two liters of LB containing ampicillin (100 µg ml^−1^) and chloramphenicol (12.5 µg ml^−1^). The cultures were grown shaking at 25°C to A_600_∼0.7 and protein synthesis was induced with IPTG (0.25 mM). Upon induction, the cultures were grown overnight at 25°C. Cells were harvested at 6000×g for 10 min at 4°C in a Avanti J-2 XPI centrifuge fitted with rotor JLA-8.1000 (Beckman Coulter). Cell pellets were re-suspended in 30 ml of cold His-bind buffer (buffer A) [*tris*(hydroxymethyl)aminomethane-HCl (Tris-HCl) buffer (50 mM, pH 8), NaCl (500 mM)], and imidazole (5 mM) containing phenylmethanesulfonylfluoride (PMSF, 1 mM). Cells were placed on ice and lysed by sonication for 2 min (2-s pulse followed by 4 s of cooling) at level 7 in a model 550 sonic dismembrator (Fisher). The extract was cleared by centrifugation at 4°C for 30 min at 43,367×*g*. H_6_-*Sl*Acs-*Se*Acs chimera was purified from clarified cell extract using a 1 ml settled bed volume of HisPur™ Ni-NTA Resin (Pierce). Unbound proteins were eluted off the column by washing with buffer A. The resin was washed with 10 column volumes of buffer B [Tris-HCl buffer (50 mM, pH 8), NaCl (500 mM), and imidazole (15 mM)]. H_6_-*Sl*Acs-*Se*Acs chimera was eluted with 5 column volumes of buffer C [Tris-HCl buffer (50 mM, pH 8), NaCl (500 mM), and imidazole (250 mM)]. All fractions containing H_6_-*Sl*Acs-*Se*Acs chimera were combined. rTEV protease was added to H_6_-*Sl*Acs-*Se*Acs chimera and the *Sl*Acs-*Se*Acs chimera/rTEV mixture was incubated at room temperature for 3 h. PMSF was added to the protein mixture and incubated 15 min at room temperature. The *Sl*Acs-*Se*Acs chimera/rTEV mixture was dialyzed at 4°C against buffer D (Tris-HCl (50 mM, pH 8), NaCl (500 mM)) twice for 3 h and again against buffer D containing imidazole (5 mM) for 12 h. After cleavage and dialysis, protein mixtures were passed over 1 ml HisPur™ Ni-NTA Resin (Pierce) using the buffers described above. Cleaved *Sl*Acs-*Se*Acs chimera passed through the resin and eluted in the flow-through fractions. Purified *Sl*Acs-*Se*Acs chimera was analyzed by SDS-PAGE. Fractions containing *Sl*Acs-*Se*Acs chimera were pooled together. *Sl*Acs-*Se*Acs chimera was stored in Tris-Cl buffer (50 mM, pH 8.0) containing NaCl (100 mM) and glycerol (20%, v/v). *Sl*Acs concentration was determined by measuring absorbance at 280 nm. The molecular weights and molar extinction coefficients used to calculate H_6_-*Sl*Acs-*Se*Acs chimera concentrations are listed in Table 3. All enzymes were purified to >95% homogeneity.

**Table 3:** Molecular mass and molar extinction coefficients of proteins used in this study.

Protein	MM (Da)	ε (M^−1 ^cm^−1^)
*Sl*Acs	71045	135455
*Se*Acs	72153	138770
A1 chimera	71527	150925
A2 chimera	71541	150925
A3 chimera	71500	150800
A4 chimera	71432	147945
A5 chimera	71352	146455
A6 chimera	71115	135455
B1 chimera	71234	139925
B2 chimera	71471	150800
B3 chimera	71471	150925
B4 chimera	71530	150925
B5 chimera	71466	150925
B6 chimera	71751	150925
C1 chimera	71297	145425
C2 chimera	71627	150925
C3 chimera	71429	145425
C4 chimera	71293	139800
C5 chimera	71104	135330
C3 chimera K609A variant	71429	145425
*Sl*PatA	108369	57760

### *Se*Acs Protein Purification

Plasmid pACS10 was transformed into a Δ*pka* derivative of *E. coli* strain C41λ(DE3) (JE9314). The resulting strain was grown overnight and sub-cultured 1∶100 (v/v) into two liters of LB containing ampicillin (100 µg ml^−1^). The culture was grown shaking at 37°C to A_600_∼0.7 and protein synthesis was induced with IPTG (0.25 mM). Upon induction, the cultures were grown overnight at 30°C. *Se*Acs was purified and stored as described [2]. *Sl*Acs^WT^ and *Sl*PatA^WT^ were purified as described [18].

### In vitro CoA Ligase Assays

Activity of *Sl*Acs^WT^, *Se*Acs^WT^, and *Sl*Acs-*Se*Acs chimera activities were measured using an NADH-consuming assay [12], [28] with modifications. Reactions (100 µl total volume) contained 4-(2-hydroxyethyl)-1-piperazineethanesulfonic acid (HEPES, 50 mM, pH 7.5), *tris*(2-carboxyethyl)phosphine (TCEP, 1 mM), ATP (2.5 mM) CoA (0.5 mM), MgCl_2_ (5 mM), KCl (1 mM), phosphoenolpyruvate (3 mM), NADH (0.1 mM), pyruvate kinase (1 U), myokinase (5 U), lactate dehydrogenase (1.5 U) and acetate (0.2 mM). Reactions were started by the addition of Acs (5–100 pmol). The absorbance at 340 nm was monitored in a 96-well plate using the Spectramax Plus UV-visible spectrophotometer (Molecular Devices). Enzyme activities were determined to be in the linear range of the assay and were calculated as described [28].

### In vitro Protein Acetylation Assay

Protein acetylation was observed using radiolabeled Ac-CoA as described [10], [12], [29]. Acetylation reactions contained 2-(*bis*(2-hydroxyethyl)imino)-2-(hydroxymethyl)-1,3-propanediol (Bis-Tris-HCl) buffer (50 mM, pH 6.0), [1-^14^C]-Ac-CoA (20 mM), acyl-CoA synthetase or acyl-CoA synthetase *C*-terminal domain (3 µM), glycerol (10%, v/v), and *Sl*PatA^WT^ (1 µM). Reactions (20 µl total volume) were incubated for 60 min at 30°C. Samples (5 µl) were resolved using SDS-PAGE [30] and proteins were visualize by Coomassie Blue R staining [31]. Gels were dried and exposed 16 h to a multipurpose phosphor screen (Packard). Labeled proteins were visualized using a Typhoon Trio+ Imager (GE Healthcare) equipped with ImageQuant TL software (GE Healthcare). Acetylation was quantified as digital light units and is reported relative to *Se*Acs^WT^ acetylation.

The effect of acetylation on activity of *Sl*Acs^WT^, *Se*Acs^WT^, and *Sl*Acs*-Se*Acs chimera activity was determined as described [12] with modifications. *Sl*Acs^WT^, *Se*Acs^WT^, or *Sl*Acs*-Se*Acs (3 µM) was incubated with *Sl*PatA^WT^ (1 µM) and 50 µM Ac-CoA for 90 min at 30°C using the buffer system described above. After 90 min, reactions were diluted into HEPES buffer (50 mM, pH 7.5 at 4°C). *Sl*Acs^WT^, *Se*Acs^WT^, and *Sl*Acs*-Se*Acs chimera activities were measured as described above.

### In vitro Deacetylation Assays

Acetylated *Sl*Acs-*Se*Acs chimera C3 was deacetylated with *S. enterica* CobB_S_ (*Se*CobB_S_) sirtuin deacetylase as described [29]. *In* *vitro* acetylated *Sl*Acs-*Se*Acs chimera C3 (3 µM, radiolabeled) was incubated with *Se*CobB_S_ (3 µM) in deacetylation buffer containing HEPES buffer (50 mM, pH 7.0), NAD^+^ (1 mM) for 60 min at 37°C (10 µl reaction volume). Reaction mixture samples (5 µl) were resolved by SDS-PAGE, and subjected to phosphor imaging analysis to assess the acetylation state of *Sl*Acs-*Se*Acs chimera C3 after incubation with *Se*CobB_S_.

H_6_-*Sl*Acs, H_6_-*Se*Acs, or H_6_-Chimera C3 enzymes isolated from *S. enterica* were deacetylated with *Se*CobB_S_ as described above with modifications. H_6_-*Sl*Acs, H_6_-*Se*Acs, or H_6_-Chimera C3 enzymes (1 mM) were incubated with *Se*CobB_S_ (1 µM) in deacetylation buffer containing HEPES (50 mM, pH 7.0), NAD^+^ (1 mM) for 60 min at 37°C (25 µl reaction volume). Acs activity was measured using the CoA synthetase assay described above.

## Results

### *S. lividans* Acetyl-CoA Synthetase (*Sl*Acs) is Functional *in vivo* in a Heterologous System

**Figure 1:**
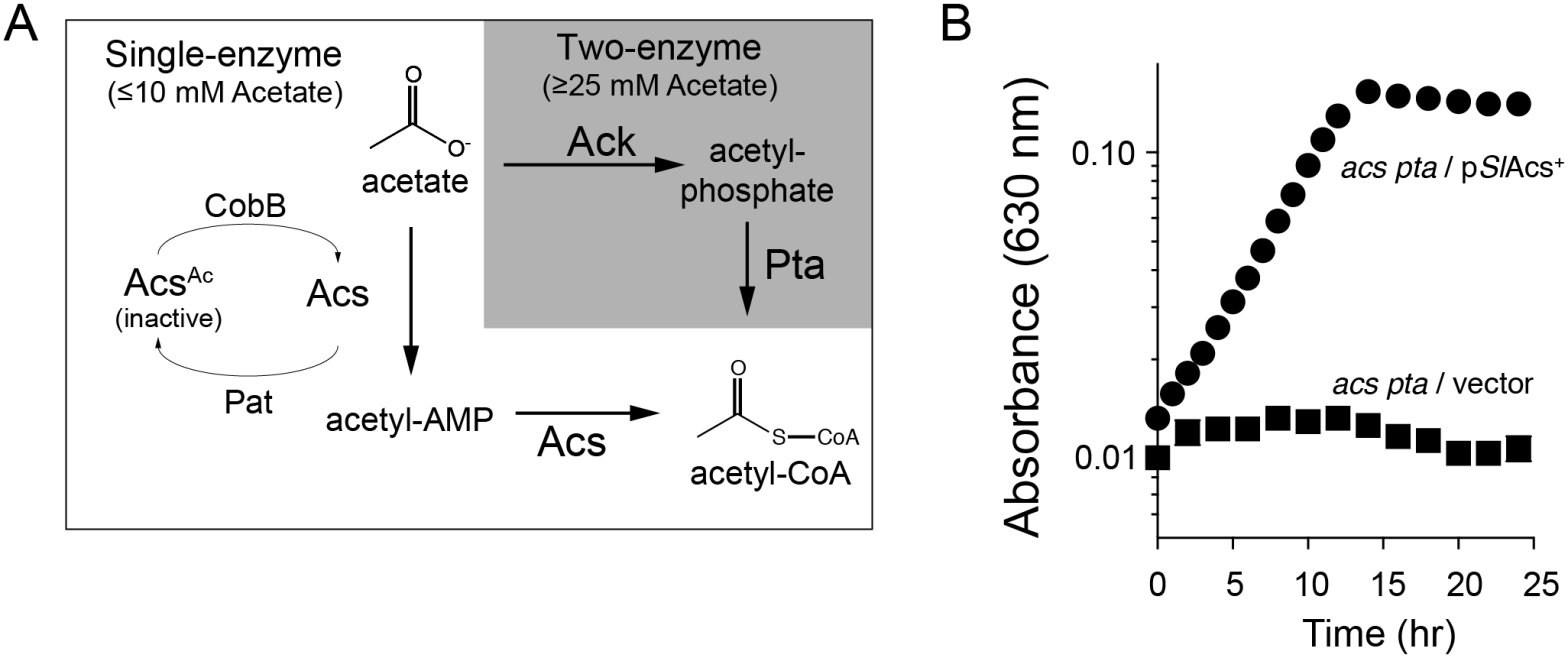
*Sl*Acs^WT^ can substitute for *Se*Acs^WT^ in *S. enterica* during growth on acetate. A. *S. enterica* encodes a one-enzyme and a two-enzyme pathway for acetate activation. The one-enzyme pathway is composed of acetyl-CoA synthetase (Acs), whose activity is modulated post-translationally by the protein acetyltransferase (Pat) and sirtuin deacetylase (CobB) enzymes. The two-enzyme pathway is comprised of acetate kinase (Ack) and phosphotransacetylase (Pta). B. Growth behavior of Δ*acs* Δ*pta S. enterica* strain JE13238 as a function of *Sl*Acs^WT^. Experiments were performed on NCE minimal medium supplemented with acetate (10 mM), at 37°C using a microtiter plate and a plate reader (Bio-Tek Instruments). Synthesis of *Sl*Acs^WT^ was ectopically encoded (plasmid p*Sl*Acs6) and induced using L-(+)-arabinose (5 mM). Cloning vector (pBAD30) lacking *S. lividans acs^+^* was used as negative control. All S.D. <0.01 absorbance units.

The *Se*Acs homologue from *S. lividans* converts acetate to acetyl-CoA *in vitro* [18]. Alignment of the *Se*Acs and *Sl*Acs amino acid sequences using BLAST revealed 52% sequence identity and 62% sequence similarity in amino acid sequence. To determine whether or not *Sl*Acs functioned *in vivo*, we expressed *S. lividans acs^+^* ectopically in a Δ*acs* Δ*pta S. enterica* strain (JE13238) demanding growth on low concentrations of acetate (10 mM). *S. enterica* uses two pathways for the conversion of acetate to acetyl-CoA (Fig. 1A) [11], [32]. One pathway is comprised of *Se*Acs, which catalyzes a two-step conversion of acetate to acetyl-CoA via an acetyl-AMP intermediate. RLA controls *Se*Acs activity [2]. The protein acetyltransferase *Se*Pat acetylates and inactivates of *Se*Acs (discussed further below) [10], and *Se*Acs is deacetylated and reactivated by the sirtuin type deacetylase *Se*CobB [2], [29]. In the second pathway, acetate kinase (Ack) and phosphotransacetylase (Pta) catalyze the conversion of acetate to acetyl-CoA via an acetyl-phosphate intermediate. Acs activity is used by the cell when the concentration of acetate in the environment is <10 mM, whilst Pta/Ack is the preferred pathway when *S. enterica* is growing on concentrations of acetate ≥25 mM. A *S. enterica* strain lacking the Acs and Ack/Pta pathways failed to grow on acetate (10 mM, Fig. 1B, squares). When *Sl*Acs was produced ectopically, growth of an *S. enterica* Δ*acs* Δ*pta* strain was restored (Fig. 1B, circles), demonstrating that *Sl*Acs was active and could substitute for *Se*Acs function *in vivo*.

### *Sl*PatA Acetylates the *C*-terminal Domain of *Se*Acs, but not *Sl*Acs

AMP-forming CoA synthetases are two-domain enzymes that activate carboxylic acids to CoA thioesters in a two-step reaction. In the first half-reaction, an invariant lysine in the *C*-terminal domain (K609 of *Se*Acs) is buried in the active site cleft located between the *N-* and *C*-terminal domains [33]. Upon adenylylation of the carboxylic acid substrate, the *C*-terminal domain undergoes a ∼140° domain rotation to allow for the thioesterification of the fatty acyl-AMP intermediate [34]. The catalytic lysine of AMP-forming CoA ligases is surface exposed when the enzyme is in the thioester-forming conformation [33], and this likely represents the conformation that is subject to acetylation by Pat.

Previously, we demonstrated that *Sl*Acs was a poor substrate for the *Sl*PatA enzyme *in vitro* [18]. That work identified *Sl*Acs as the first example of an acetyl-CoA synthetase that was not recognized by the cognate Pat protein acetyltransferase *in vitro* [10], [12]. However, *Sl*PatA efficiently acetylated and inactivated the acetoacetyl-CoA synthetase *Sl*AacS from *S. lividans*, and the orthologous *Se*Acs enzyme [18], indicating that *Sl*PatA was catalytically active, but somehow unable to acetylate *Sl*Acs *in vitro*.

We considered the possibility that *Sl*Acs favored the adenylyation conformation *in vitro*, which would likely render the target K610 inaccessible to *Sl*PatA due to its location in the *Sl*Acs active site. To differentiate the inaccessibility of *Sl*Acs K610 from the inability of *Sl*PatA to recognize *Sl*Acs, we isolated the *C*-terminal domains of *Se*Acs (a good substrate of *Sl*PatA) and *Sl*Acs. In the absence of the *N*-terminal domain, the target lysine is no longer protected, thus it is accessible to the acetyltransferase.

**Figure 2:**
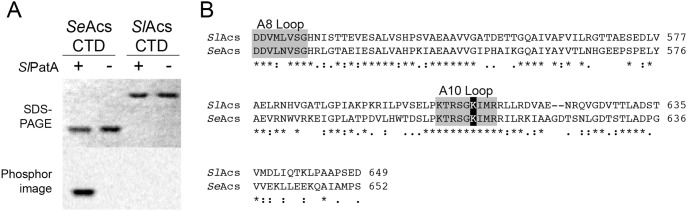
*Sl*PatA efficiently acetylates the *C*-terminal domain of *Se*Acs. A. The *C*-terminal domain of *Sl*Acs^WT^ or *Se*Acs^WT^ was incubated with [1-^14^C]-acetyl-CoA in the presence or absence of *Sl*PatA^WT^. Proteins were separated by SDS-PAGE and stained with Coomassie Blue R to visualize proteins. Acetylation was visualized by phosphor imaging. B. Alignment of the *C*-terminal domain of *Sl*Acs and *Se*Acs. “ * ” denotes conserved residues; “.” denotes similar residues; light gray boxes denote conserved loops of the AMP-forming CoA ligase family [39]; dark gray box denotes catalytic lysine.

Homogeneous *C*-terminal domains of *Sl*Acs (residues D519–D649, 131 aa) and *Se*Acs (residues D518-S652, 135 aa) were incubated in the presence of *Sl*PatA and radiolabeled [1-^14^C] acetyl-CoA. Differential migration of the *C*-terminal domains is likely due to differences in hydropathy of (grand average of hydropathy [GRAVY] scores [35] for *Sl*Acs and *Se*Acs *C*-terminal domains are +0.023 and −0.160, respectively), which has been shown to affect gel mobility of protein in SDS-PAGE [36]. As shown in figure 2A, the *C*-terminal domain of *Se*Acs was acetylated, but the *Sl*Acs *C*-terminal domain was not. These data showed that the *N*-terminal domain of *Se*Acs was not required for acetylation by *Sl*PatA. Additionally, these results strongly suggested that inaccessibility of residue K610 was likely not the reason why *Sl*Acs was poorly acetylated *in vitro*. We hypothesized that regions within the *C*-terminal domain of *Sl*Acs enzyme prevented acetylation of *Sl*PatA. As shown in figure 2B, the *C*-terminal domains of *Sl*Acs and *Se*Acs share ∼50% sequence identity.

### Chimeras of *Sl*Acs and *Se*Acs Reveal Regions in the *Se*Acs C-terminal Domain that are Critical for Acetylation by *Sl*PatA

**Figure 3:**
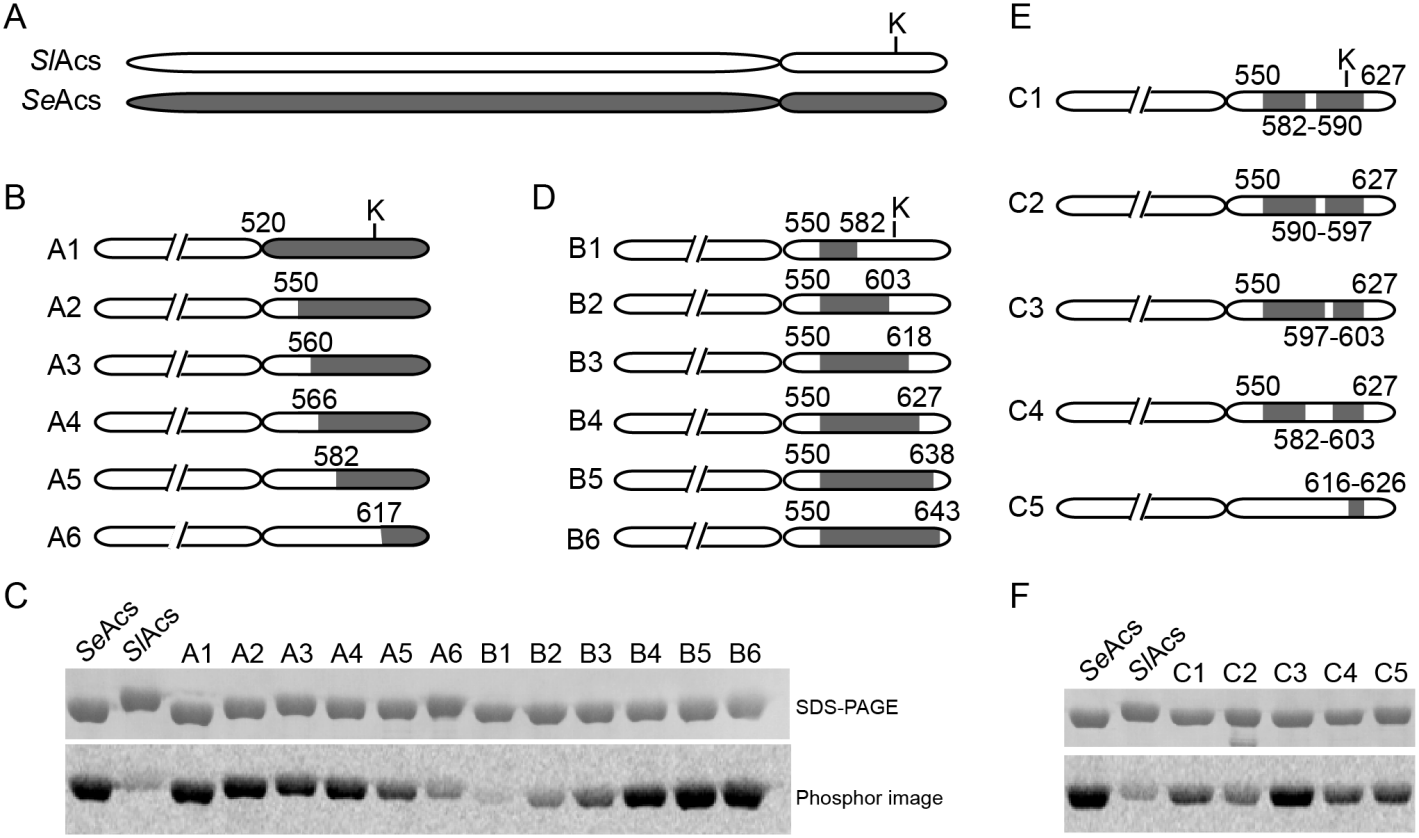
Construction and acetylation of *Sl*Acs-*Se*Acs chimeras. A. A scheme of *Sl*Acs^WT^ (white) and *Se*Acs^WT^ (grey) drawn to scale. Target lysine K610 for *Sl*Acs^WT^ and K609 aligned and depicted by “K”. The *N*-terminal domains (520 residues) are shortened with a hatch in all remaining panels. B. Schematic representation of *Sl*Acs-*Se*Acs chimeras A1–A6 in which the *C*-terminal portion of *Sl*Acs^WT^ (white) was replaced with the corresponding amino acid sequence from *Se*Acs^WT^ (gray). All chimeras are drawn to scale for reference to the target lysine (denoted by “K”). Numbers all denote the fusion points with respect to the *Sl*Acs protein sequence (i.e. either the first residue of *Sl*Acs^WT^ replaced by *Se*Acs^WT^ sequence or the first residue of *Sl*Acs^WT^ after the *Se*Acs^WT^ amino acid sequence). C. Acetylation of *Sl*Acs-*Se*Acs chimeras A1–A6 and B1–B6 using *Sl*PatA^WT^ and [1-^14^C]acetyl-CoA. D. Schematic or *Sl*Acs-*Se*Acs chimers B1–B6 in which internal portion of the *C*-terminal *Sl*Acs domain are replaced with the corresponding sequence from *Se*Acs^WT^. E. Schematic of chimeras C1–C5. F. Acetylation of *Sl*Acs-*Se*Acs chimeras C1–C6.

Based on regions of sequence conservation (Fig. 2B), we generated a set of precise fusions between the *Sl*Acs and *Se*Acs that contained varying amounts of the *Se*Acs protein. A *Sl*Acs chimera containing the *Sl*Acs *N*-terminal domain fused to the *Se*Acs *C*-terminal (chimera A1) was strongly acetylated by *Sl*PatA, confirming that the *C*-terminal domain of *Sl*Acs was responsible for the poor acetylation of *Sl*Acs^WT^ (Figs. 3A, B).

We identified regions of the *Se*Acs *C*-terminal domain important for acetylation by *Sl*PatA by constructing chimeras that contained decreasing amounts of the *Se*Acs *C-*terminal domain relative to chimera A1. To measure the efficiency of acetylation, each chimera was incubated with *Sl*PatA and radiolabeled [1-^14^C] acetyl-CoA. *Sl*PatA strongly acetylated chimeras containing at least the final 86 amino acids of *Se*Acs (chimeras A1, A2, A3, A4; Fig. 3). These chimeras contained at least 43 amino acids *N*-terminal to the acetylation site, a region previously reported to be important for acetylation of homologous AMP-forming CoA ligase enzymes by the *R. palustris* Pat homologue [17].

To narrow down the number of *Se*Acs residues required for acetylation of the *Sl*Acs*-Se*Acs chimeras, we focused on *Sl*Acs*-Se*Acs chimera A2, which had the fewest *Se*Acs-derived residues (Fig. 3B), and the highest level of acetylation (Fig. 3C).

We generated a second set of chimeras in which various stretches of *Sl*Acs-derived residues were substituted into *Sl*Acs*-Se*Acs chimera A2 (Fig. 3D). *Sl*Acs*-Se*Acs chimeras B4, B5, and B6 that contained at least 45 residues of *Se*Acs (including the *Se*Acs^K609^ acetylation site) were strongly acetylated (Fig. 3C). Notably, the A10 loop of Acs, which contains the target lysine, is completely conserved between *Se*Acs and *Sl*Acs (Fig. 2B). However, 17 amino acids *C*-terminal to the acetylation site of *Se*Acs were required for acetylation by *Sl*PatA. This revealed a previously unrecognized region of the protein important for acetylation. Of this set of *Sl*Acs-*Se*Acs chimeras, chimera B4 was the best substrate of *Sl*PatA and contained the fewest *Se*Acs-derived amino acids.

**Figure 4:**
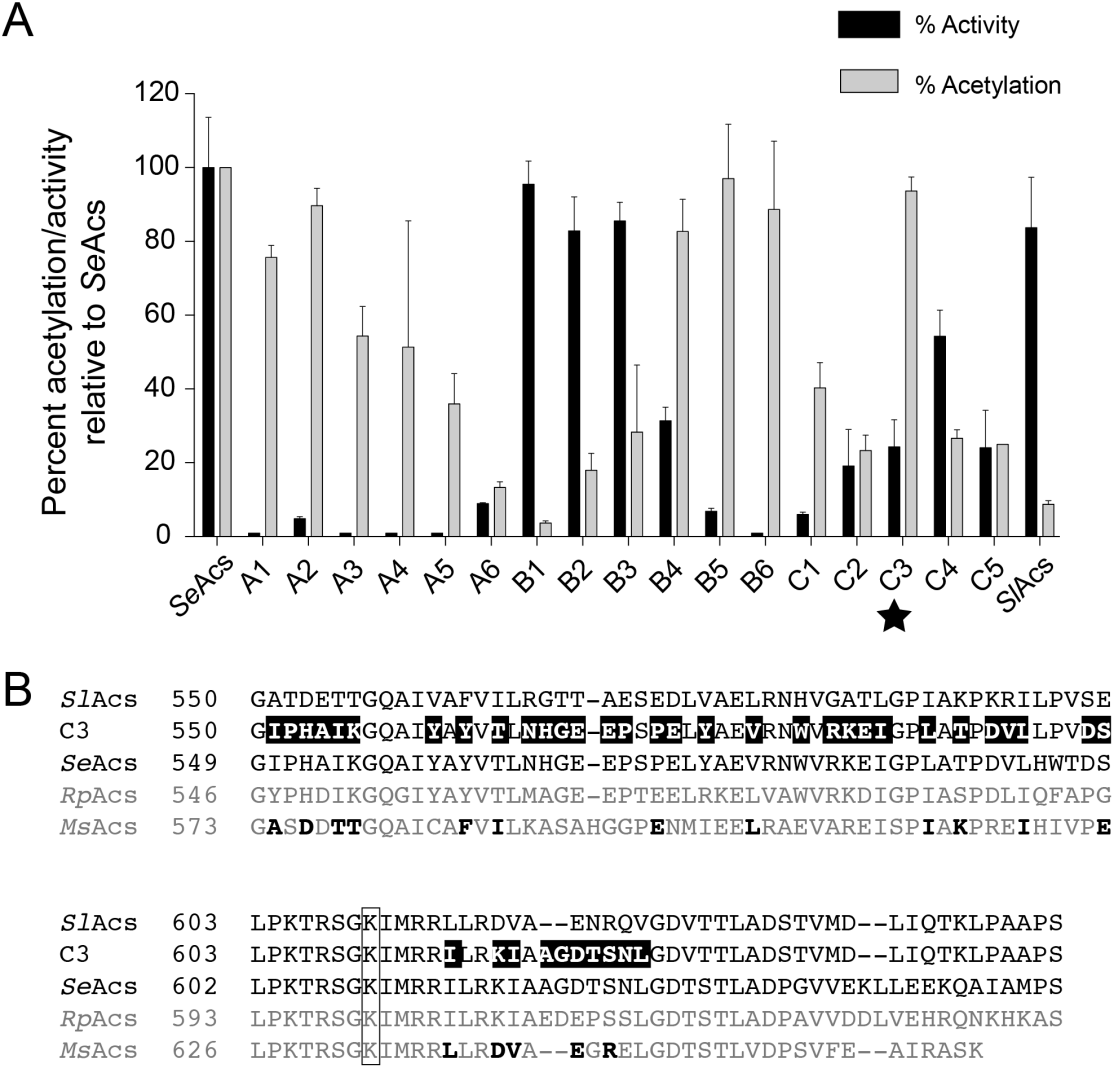
*Sl*Acs-*Se*Acs Chimera C3 is active and efficiently acetylated. A. Acetyl-CoA synthetase activity of each chimera and *Sl*Acs^WT^ relative to *Se*Acs^WT^ (gray bars). Amount of acetylation in figure 3C and 3F was quantified and normalized to the total acetylation of *Se*Acs (black bars). *Sl*Acs-*Se*Acs chimera C3, the most efficiently acetylated, active chimera with the fewest *Se*Acs^WT^-derived residues, is noted with a star. Values are reported as the mean ± S.D. of three experiments. B. Sequence alignment of *Sl*Acs^WT^, *Se*Acs^WT^, chimera C3, *Rhodopseudomonas palustris* CGA009 Acs (*Rp*Acs), and *Mycobacterium smegmatis* mc^2^155 Acs (*Ms*Acs). Residues in chimera C3 that are derived from the *Se*Acs^WT^ amino acid sequence are highlighted in black. *Sl*Acs residues conserved in the *Ms*Acs homologue are shown in bold typeface in the sequence of the latter. Black box indicates the target lysine.

To determine whether the 77 contiguous *Se*Acs-derived residues of chimera B4 were critical for acetylation, we identified regions of *Sl*Acs and *Se*Acs with low amino acid sequence conservation and introduced those sets of *Sl*Acs residues into chimera B4 (Fig. 3E). Of those tested, only *Sl*Acs-*Se*Acs chimera C3 was acetylated with similar efficiency as *Se*Acs (Fig. 3F). Acetylation of each chimera was quantified relative to acetylation of *Se*Acs (Fig. 4A, gray bars).

Importantly, chimeras containing only the 60 *Se*Acs-derived residues *N*-terminal to K610 (chimera B2) or 11 *Se*Acs-derived residues *C*-terminal to K610 (chimera C5) were <30% acetylated relative to *Se*Acs. Thus, amino acid sequences *N*- and *C*-terminal to the target lysine were important for acetylation by *Sl*PatA, and neither set of amino acids rendered *Sl*Acs a strong acetylation target when introduced independently.

### Assessment of the Enzymatic Activity of the Chimeras

Chimeras were tested for their AMP-forming acetyl-CoA ligase forming activity. Although the *Sl*Acs^WT^ and *Se*Acs^WT^ *C*-terminal domains share a high degree of sequence conservation, not all chimeras were active (Fig. 4A, black bars). To identify active chimeras that were also targets of acetylation, the acetylation of each chimera was measured relative to *Se*Acs (Fig. 4A, gray bars). *Sl*Acs-*Se*Acs chimera C3 (hereafter referred to as chimera C3) was identified as the single chimera with the fewest *Se*Acs residues that was active and efficiently acetylated by *Sl*PatA. As shown in figure 4B, chimera C3 contained 41 amino acid differences from *Sl*Acs. For comparison, we include the analogous sequence from Acs homologues known to acetylated by protein acetyltransferases in other bacteria. Notably, the wildtype *Sl*Acs amino acid sequence replaced by *Se*Acs sequences shares some sequence homology with these Acs homologues.

### Chimera C3 Activity is Modulated by Acetylation and Deacetylation

**Figure 5:**
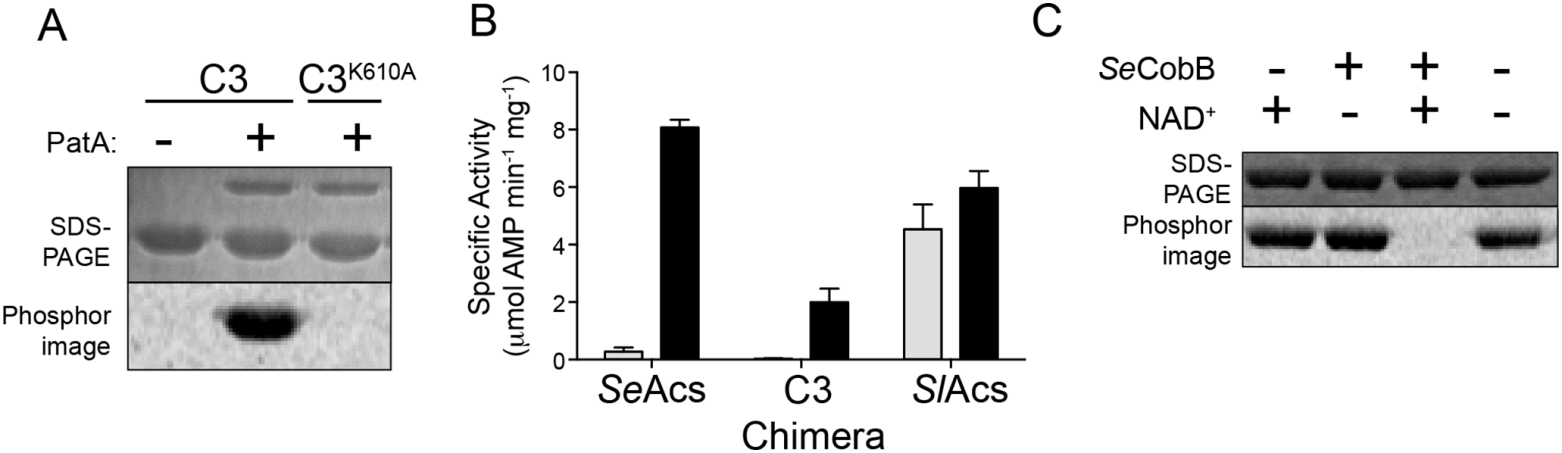
Chimera C3 is regulated by reversible lysine acetylation. A. Chimera C3 or chimera C3^K610A^ was incubated with [1-^14^C]-acetyl-CoA in the presence or absence of *Sl*PatA^WT^. Proteins were separated by SDS-PAGE and stained with Coomassie Blue R to visualize proteins. Acetylation was visualized by phosphor imaging. B. Chimera C3, *Se*Acs^WT^, or *Sl*Acs^WT^ was incubated in the presence (grey bars) or absence (black bars) of *Sl*PatA^WT^. Samples were removed, diluted, and assayed to measure acetyl-CoA synthetase activity after 90 min incubation with *Sl*PatA^WT^. Acs activity was measured in an NADH-consumption assay. Values are reported as the mean ± S.D. of three experiments. C. Chimera C3 previously acetylated by *Sl*PatA^WT^ with [1-^14^C]-acetyl-CoA was incubated with the addition of *Se*CobB^WT^ and/or NAD^+^. Proteins were resolved by SDS-PAGE and stained with Coomassie Blue R to visualize proteins. Acetylation was visualized by phosphor image.

As shown in figure 5A, the catalytic residue K610 residue is the only residue of chimera C3 that was acetylated. To test whether the activity of chimera C3 was under the control of acetylation, the protein was incubated with *Sl*PatA acetyltransferase in the presence and absence of the acetyl donor, acetyl-CoA. Upon acetylation, chimera C3 activity decreased >98%, similar to the regulation of *Se*Acs activity (Fig. 5B, gray bar). The *Sl*Acs enzyme retains >75% activity upon incubation with *Sl*PatA and Ac-CoA (Fig. 5B, gray bar). As mentioned above, acetylation of *Se*Acs^WT^ is reversed by the NAD^+^-dependent sirtuin deacetylase CobB in *S. enterica,* and deacetylation reactivates the *Se*Acs^WT^ enzyme [2]. We tested whether chimera C3 could be deacetylated by incubating acetylated chimera C3 with *Se*CobB, the co-substrate NAD^+^, or both. When *Se*CobB and NAD^+^ were present in the reaction mixture, chimera C3^Ac^ was completely deacetylated (Fig. 5C), demonstrating that the reversibility of the acetylation process was not affected by the substitutions in chimera C3.

### *Sl*Acs-*Se*Acs Chimera C3 is Acetylated *in vivo* in *S. enterica* by *Sl*patA

**Figure 6:**
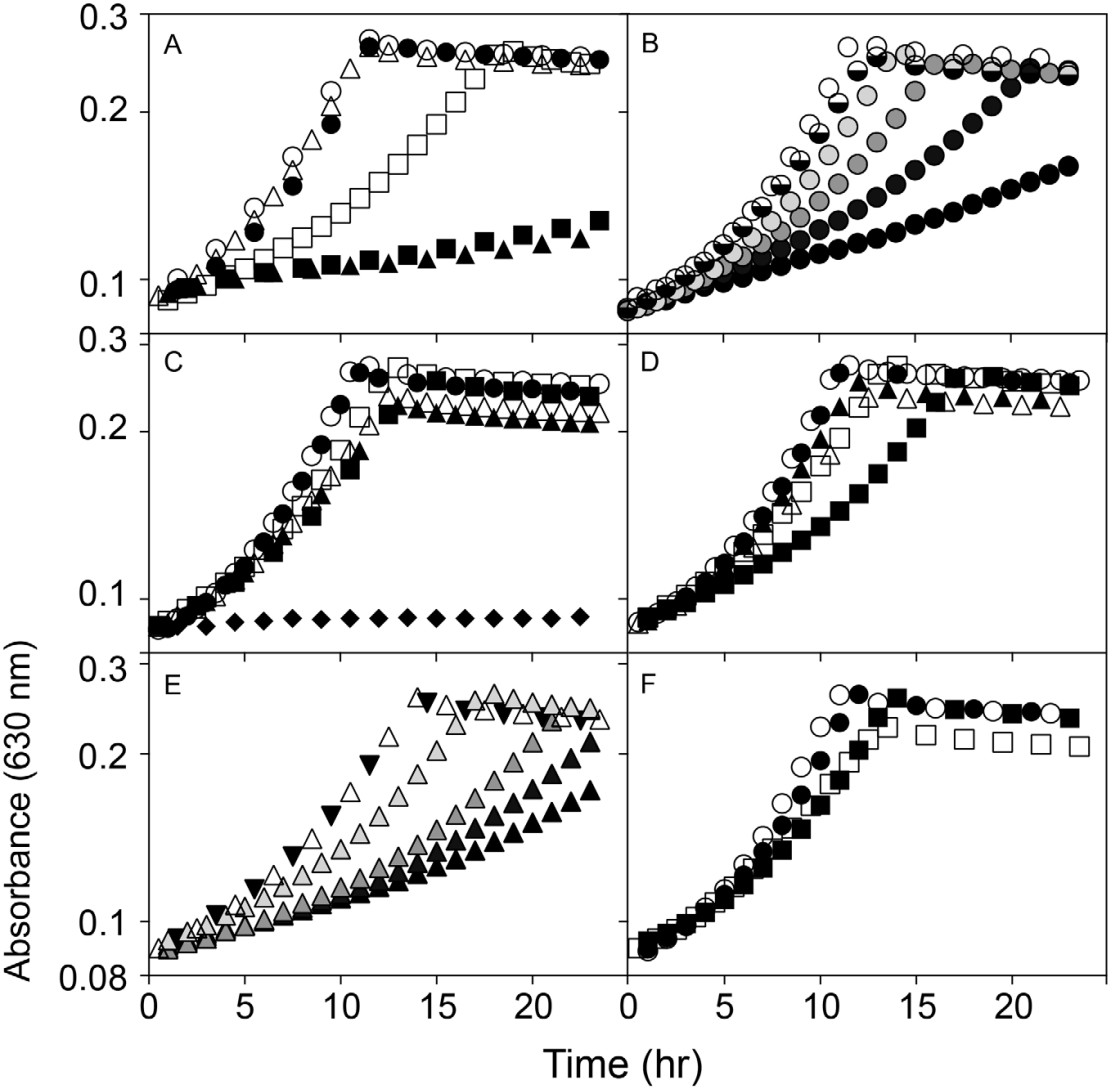
Chimera C3 is regulated by *Sl*PatA in vivo in *S. enterica*. Growth behavior of *S. enterica* in NCE minimal medium supplemented with acetate (10 mM). A. Growth of *S. enterica* Δ*acs pat* Δ*cobB* producing H_6_-*Se*Acs^WT^ (triangles), H_6_-*Sl*Acs^WT^ (circles), or H_6_-Chimera C3 (squares) harboring either a plasmid expressing *Sl*PatA^WT^ (filled shapes) or an empty vector (open shapes). All media was supplemented with 25 µM IPTG. B. Growth of *S. enterica* Δacs *pat* Δ*cobB* (JE9152) producing H_6_-*Sl*Acs^WT^ harboring a plasmid producing *Sl*PatA^WT^ induced with IPTG concentrations of 25 µM (open circles), 50 µM (light gray), 100 µM (medium gray), 250 µM (dark gray), or 500 µM (black). For reference, half-filled circles denote an equivalent strain producing H_6_-*Sl*Acs^WT^ harboring an empty vector induced with 500 µM IPTG. C. Growth of *S. enterica* Δ*acs pat* Δ*cobB* (JE9152) producing H_6_-*Se*Acs^WT^ (triangles), H_6_-*Sl*Acs^WT^ (circles), H_6_-Chimera C3 (squares), or empty vector (diamonds) harboring either a plasmid expressing *Sl*PatA^WT^ (filled shapes) or an empty vector (open shapes). All media was supplemented with 5 µM IPTG. D. Growth of *S. enterica* Δ*acs pat cobB* ^+^ (JE9894) producing H_6_-*Se*Acs^WT^ (triangles), H_6_-*Sl*Acs^WT^ (circles), or H_6_-Chimera C3 (squares) harboring either a plasmid expressing *Sl*PatA^WT^ (filled shapes) or an empty vector (open shapes). All media was supplemented with 25 µM IPTG. E. Growth of *S. enterica* Δ*acs pat cobB* ^+^ (JE9894) producing H_6_-*Sl*Acs-*Se*Acs chimera C3 harboring a plasmid producing *Sl*PatA^WT^ induced with IPTG concentrations of 10 µM (open triangle), 25 µM (light gray), 500 µM (medium gray), 100 µM (dark gray), or 250 µM (black). For reference, the inverted, filled triangles denote the growth of an equivalent strain producing H_6_-*Sl*Acs-*Se*Acs chimera C3 harboring an empty vector (no *Sl*PatA^WT^) induced with 500 µM IPTG. F. *S. enterica* Δ*acs pat cobB* ^+^ strains (JE9894) producing H_6_-*Se*Acs^WT^ (circles) or H_6_-*Sl*Acs^WT^ (squares) are shown growing with high induction (250 µM IPTG) of empty vector control (open symbols) or a plasmids expressing *Sl*PatA. F. All S.D. <0.015 absorbance units.

To determine the efficiency of *Sl*PatA acetylation of chimera C3 *in vivo*, we used *S. enterica* acetate utilization (Fig. 1A) as a heterologous model to demonstrate the effects of *Sl*PatA acetylation on activity of the Acs homologues. In this system, His-tagged chimera C3, *Sl*Acs, and *Se*Acs (H_6_-chimera C3, H_6_-*Sl*Acs, H_6_-*Se*Acs, respectively) were produced from plasmids in *S. enterica acs pat cobB* and *S. enterica acs pat cobB* ^+^ strains JE9152 and JE9894, respectively. All the experiments were conducted in *S. enterica pat* strains to prevent acetylation by *Se*Pat. We characterized the effect of *Sl*PatA acetylation on the H_6_-Acs homologues by measuring growth of each strain harboring a plasmid with an inducible *Sl*PatA allele or an empty cloning vector. Additionally, we isolated H_6_-*Se*Acs^WT^, H_6_-*Sl*Acs^WT^, and H_6_-chimera C3 from cells grown in the presence or absence of *Sl*PatA to quantify the effects of *Sl*PatA acetylation on each Acs protein. As shown in figure 6A, production of H_6_-chimera C3, H_6_-*Sl*Acs, or H_6_-*Se*Acs supported growth of *S. enterica acs pat cobB* strain (open symbols). This was the expected result, since the strain lacked Pat activity, thus the cell could not acetylate (i.e., inactivate) any of the Acs enzymes. We attributed the lag in the strain producing H_6_-chimera C3 to the decreased activity of this chimera (Fig. 5B). When production of *Sl*PatA was induced in each strain (25 µM inducer), growth of *S. enterica acs pat cobB* strains producing H_6_-*Se*Acs^WT^ or H_6_-chimera C3 was significantly reduced, while growth of the *S. enterica acs pat cobB* strain producing H_6_-*Sl*Acs^WT^ was unaffected. Importantly, inhibition of an *S. enterica acs cobB* strain producing H_6_-*Sl*Acs^WT^ required high levels of *Sl*PatA^WT^ induction (500 µM inducer, Fig. 6B). No growth inhibition occurred when *Sl*PatA^WT^ was induced at low levels (≤5 µM inducer, Fig. 6C).

As expected, the presence of *Se*CobB^WT^ in a *S. enterica acs pat cobB^+^* strain resulted in no significant growth defects upon *Sl*PatA^WT^ induction in strains expressing H_6_-*Sl*Acs^WT^ or H_6_-*Se*Acs^WT^ (Fig. 6D). However, we did note a slight inhibition of growth of a *S. enterica acs pat cobB^+^* strain producing H_6_-chimera C3. We surmised that such an effect was likely due to a decreased ability of *Se*CobB^WT^ to deacetylate and reactivate H_6_-chimera C3 and restore growth. This idea was supported by the observation that increased induction of *Sl*PatA^WT^ inhibited a *S. enterica acs pat cobB^+^* strain producing H_6_-chimera C3 (Fig. 6E), but not those producing H_6_-*Sl*Acs^WT^ nor H_6_-*Se*Acs^WT^ (Fig. 6F).

Since high levels of *Sl*PatA induction were required to inhibit growth of an *S. enterica acs cobB* strain producing H_6_-*Sl*Acs^WT^, we expected that H_6_-*Sl*Acs^WT^ to be poorly acetylated by *Sl*PatA^WT^ and thus more active *in vivo*. We also expected higher proportions of acetylated to non-acetylated H_6_-*Se*Acs^WT^ and H_6_-chimera C3 *in vivo*. To measure the effect of *Sl*PatA^WT^ acetylation on the activity of H_6_-*Sl*Acs of, H_6_- *Se*Acs of, and H_6_-chimera C3, we grew *S. enterica acs cobB* strains expressing the corresponding *acs* alleles while inducing *Sl*PatA^WT^ at low levels (5 µM) to allow for growth and biomass accumulation for all strains (Fig. 6C). H_6_-*Sl*Acs^WT^, H_6_-*Se*Acs^WT^ and H_6_-chimera C3 enzymes were isolated from strains harboring plasmid-borne *Sl*PatA^WT^ or empty vector.

**Figure 7:**
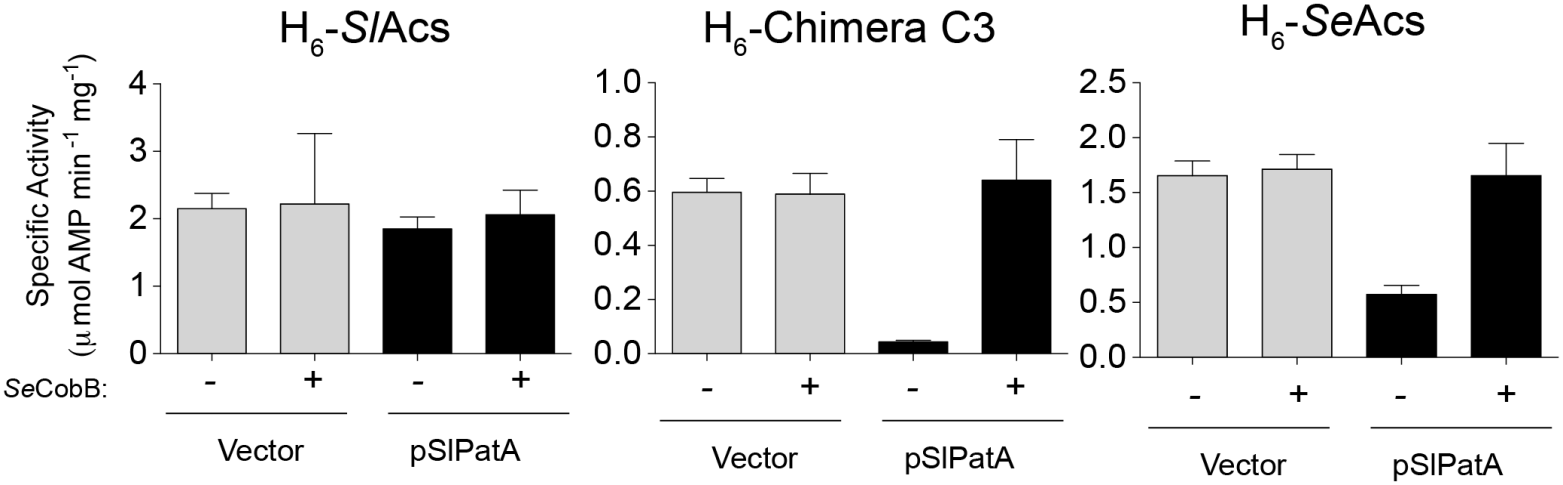
Activities of Chimera C3 and *Se*Acs^WT^ are reduced in strains expressing *Sl*PatA^WT^. H_6_-Chimera C3, H_6_-*Sl*Acs, and H_6_-*Se*Acs were produced in *S. enterica* Δ*acs pat* Δ*cobB* strain JE9152 harboring either a plasmid producing *Sl*PatA^WT^ or an empty vector. Strains were grown in NCE minimal medium supplemented with acetate (10 mM). Acs proteins were incubated in the presence or absence of *Se*CobB deacetylase and its co-substrate NAD^+^. Acs activity was measured in an NADH-consumption assay. Values are reported as the mean ± S.D. of three activity measurements.

As shown in figure 7, activity of the H_6_-*Sl*Acs^WT^ enzyme isolated from a strain producing *Sl*PatA^WT^ was not significantly reduced compared to H_6_-*Sl*Acs^WT^ isolated from a strain with no *Sl*PatA^WT^. However, activities of the H_6_-*Se*Acs^WT^ and H_6_-chimera C3 enzymes were significantly lower when isolated from strains expressing *Sl*PatA^WT^ compared to those with no *Sl*PatA^WT^. Activities of the *Se*Acs and H_6_-chimera C3 were restored upon incubation with *Se*CobB deacetylase. These data suggested that *Sl*PatA^WT^ more efficiently acetylated H_6_-*Se*Acs^WT^ and H_6_-chimera C3 than it did H_6_-*Sl*Acs in a heterologous *in vivo* model.

## Discussion

Herein we report the first Acs enzyme that is not a substrate of Pat homologues *in vitro*. This finding is important, since Acs is the paradigm for the analysis RLA in all metabolic systems reported thus far. Our results begin to shed some light onto why the *Sl*Acs is not efficiently acetylated by the *Sl*PatA^WT^ enzyme of *S. lividans*. By constructing chimeras of *Sl*Acs that are acetylated by *Sl*Pat^WT^ and retain biological activity we gained insights into structural, physiological and possibly evolutionary questions raised by this work.

### Is Acs Activity under RLA Control in Streptomycetes?

At present, the answer to this question is unclear. It is not known whether *Sl*Acs^WT^ is a *bona fide* substrate of *Sl*PatA^WT^ *in vivo* in *S. lividans.* The literature adds to the challenge of determining whether or not in streptomycetes Acs is under RLA control. Work performed by others in *Streptomyces coelicolor* suggested that the Acs enzyme of this actinomycete may be under RLA control, because results of *in vitro* experiments showed that acetylated *Sc*Acs was a substrate of a sirtuin deacetylase present in that bacterium [16]. The same authors also reported the isolation of acetylated *Sc*Acs from *S. coelicolor.* Since the *S. coelicolor* genome contains a gene encoding a *Sl*PatA homologue, they concluded that *Sc*Acs was under RLA control.

Our initial work with the *S. lividans Sl*PatA^WT^ and *Sl*Acs^WT^ enzymes paints a complex picture for the regulation of *Sl*Acs^WT^ function in this organism, and by extrapolation, maybe in *S. coelicolor*. Because the specific activity of *Sl*Acs^WT^ is similar to that of *Se*Acs^WT^ *in vitro* (Fig. 5B), we hypothesize that *Sl*Acs^WT^ activity is also tightly controlled by *S. lividans*. To account for the inability of *Sl*PatA^WT^ to acetylate *Sl*Acs^WT^, we propose that *Sl*PatA^WT^ has evolved unique strategies for substrate recognition, or *Sl*PatA^WT^ is not the primary modifier of *Sl*Acs^WT^. We discuss each possibility further below.

### *In vitro*, *Sl*PatA^WT^ does not Recognize *Sl*Acs^WT^

Pat homologues acetylate Acs in *R. palustris* and *S. enterica* [10], [12]. Clearly, acetylation of *Sl*Acs^WT^ by *Sl*PatA^WT^ does not occur efficiently *in vitro* or in a heterologous model system (Figs. 4B, 5A, 6A, 7) [18]. The following possibilities should be taken into consideration when thinking about the potential regulation of *Sl*Acs^WT^ by RLA. First, it is possible that *Sl*Acs^WT^ may have evolved to evade acetylation by *Sl*PatA^WT^. Secondly, since *S. lividans* encodes ∼72 predicted GNAT-type acetyltransferases (Pfam00583) it is possible that one of these GNATs, not *Sl*PatA^WT^, acetylates *Sl*Acs^WT^. If a GNAT other than *Sl*PatA acetylated *Sl*Acs, it begs the questions of what selective pressure drove the conformational change *Sl*Acs to avoid recognition by *Sl*PatA, and what the physiological benefits of such a change are. And thirdly, the reversed domain organization of *Sl*PatA, relative to *Rp*Pat and *Se*Pat, may prevent recognition of *Sl*Acs^WT^ by *Sl*PatA^WT^.

### Substantial Changes in the *C*-terminal Domain of *Sl*Acs^WT^ Lead to its Recognition by *Sl*PatA^WT^

Forty-one amino acid changes in the *C*-terminal domain of *Sl*Acs^WT^ were needed to allow *Sl*Pat^WT^ to recognize and acetylate *Sl*Acs (Fig. 3). If we assumed that the domain organization of *Sl*Pat^WT^ was not a factor in *Sl*Acs^WT^ recognition, such a large number of substitutions would suggest that the protein underwent dramatic evolutionary changes to prevent modification by *Sl*Pat^WT^. Importantly, we note that some *Sl*Acs sequences that were replaced in the C3 chimera exhibit homology to Acs homologues that are acetylated by GNAT enzymes in other bacteria (Fig. 4B). This suggests acetylation of Acs and other AMP-forming acyl-CoA synthetases cannot be predicted by amino acid sequence [17]. Our results indicate, however, that *Sl*Acs recognition by *Sl*PatA^WT^ is reversible by mutation, and that the resulting *Sl*Acs variant can be reversibly acetylated.

### How do Changes in the *C*-terminal Domain of *Sl*Acs Affect its Acetylation and Activity?

Studies of *R. palustris* Pat (*Rp*Pat) substrate specificity indicate that this enzyme recognizes a loop >20 residues *N*-terminal to the target lysine in the substrate protein, suggesting that the *Rp*Pat interacts with a relatively large surface of substrate proteins [17]. Here, we demonstrate that the identities of residues ranging from 8–52 amino acids *N*-terminal to the target lysine of *Se*Acs^WT^, in combination with 5–17 amino acids *C*-terminal to the target lysine of *Se*Acs^WT^ are critical for recognition of this substrate by *Sl*PatA^WT^ in isolation. This indicates that *Sl*PatA^WT^ recognizes several regions of the *Se*Acs *C*-terminal domain including the target lysine, residues *N*-terminal to the target lysine, and residues *C*-terminal to the target lysine. It is possible that these regions of the *Se*Acs *C*-terminal domain are necessary for direct interactions with the *Sl*PatA protein. Alternatively, these regions may be necessary to position the target lysine for entry into the *Sl*PatA active site. The crystal structure of *Sl*PatA^WT^ substrate *Se*Acs^WT^ is known (PDB 1PG3, 1PG4) [33]. Comparison of this structure with the structure of *Sl*Acs (structure not known) may distinguish these possibilities. Efforts to obtain the crystal structure of *Sl*Acs are ongoing.

### Is *Sl*Acs^WT^ Regulated by One or More Protein Acetyltransferases?

As mentioned above, *Sl*Acs^WT^ may have evolved to evade acetylation specifically by *Sl*PatA^WT^. However *Sl*Acs^WT^ may be acetylated *in vivo* by one of the additional 72 predicted GNAT-type acetyltransferases (Pfam00583) encoded by the genome of this bacterium or by an enzyme independent pathway. The possibility that an alternative GNAT acetylates *Sl*Acs^WT^ more efficiently than *Sl*PatA^WT^ does is not unprecedented. It is known that the genome of *R. palustris* encodes a Pat homologue and a single-domain GNAT protein acetyltransferase that share overlapping protein acetyltransferase substrates, and that both enzymes acetylate these substrates with different affinities [13]. Alternatively, *Sl*Acs^WT^ may be acetylated directly and non-enzymatic by the reactive metabolite acetyl-phosphate. This phenomenon has been characterized in *E. coli* and has been shown to affect the activity of the target enzymes [37], [38]. Therefore, the possibility of *Sl*PatA^WT^ not being the sole regulator of *Sl*Acs^WT^ activity in *S. lividans* needs to be further investigated.

### Does the Unique Domain Organization of *Sl*PatA^WT^ Affect Substrate Specificity?

Pat acetyltransferases are two-domain enzymes composed of a GNAT (acetyltransferase) domain and a large domain whose function is likely regulatory. In *Sl*PatA^WT^, the GNAT domain is located at the *N*-terminus of the protein [18]. In contrast, in *R. palustris* and *S. enterica*, the domain order is reversed (i.e., GNAT domain is at the *C*-terminus of protein). *Sl*PatA^WT^ also has a collagen-like Gly-Pro-Ser motif in the large domain [18]. *S. enterica* and *R. palustris* Pat homologues efficiently acetylate their cognate Acs enzymes *in vitro* [10], [12]. The alternate domain organization of *Sl*PatA^WT^ may account for the poor acetylation of *Sl*Acs^WT^ compared to *Se*Acs^WT^ and *Sl*AacS^WT^ *in vitro* [18]. If *Sl*PatA^WT^ has evolved strategies for recognition of protein substrates differently from *Se*Pat and *Rp*Pat, our *in vitro* assay may be missing a factor that promotes efficient *Sl*PatA^WT^ recognition of *Sl*Acs^WT^ such as a small molecule, macromolecule (*e.g.* protein), or an as-yet-unidentified intracellular condition. If this were the case, the amino acid changes introduced into *Sl*Acs^WT^ to generate the *Sl*Acs-*Se*Acs chimera C3 obviate the need for additional factors or conditions for *Sl*PatA^WT^ recognition.
